# The Potential of Ambroxol as a Panacea for Neonatal Diseases: A Scoping Review

**DOI:** 10.7759/cureus.67977

**Published:** 2024-08-27

**Authors:** Sameer Y Al-Abdi, Maryam Al-Aamri

**Affiliations:** 1 Pediatrics/Neonatology, Al-Ahsa Hospital, Al-Ahsa, SAU; 2 Pediatric Nephrology, Maternity and Children Hospital Al-Ahsa, Al-Ahsa, SAU

**Keywords:** ambroxol, meconium aspiration syndrome (mas), neonatal hypoxia, convulsion, premature chronic lung disease, intraventricular hemorrhage (ivh), patent ductus arteriosus closure, respiratory distress syndrome of prematurity, preterm neonate

## Abstract

Ambroxol, a commonly used mucolytic agent, has been extensively studied for its clinical effectiveness in managing respiratory conditions in pediatric and adult patients. The existing body of research on ambroxol demonstrates its safety and efficacy. However, its potential role in preventing and treating neonatal diseases still needs to be explored. This scoping review aims to shed light on the unexplored potential of ambroxol, particularly its applications in perinatal and neonatal care. We aim to offer valuable insights for healthcare professionals, researchers, and academics, thus presenting a positive perspective.

Key scientific databases such as Google Scholar, PubMed, Cochrane Library, and Europe PMC were meticulously searched for relevant literature on ambroxol in perinatal and neonatal medicine. Gray literature was also surveyed, and the search encompassed all study designs and languages up to June 2024. Furthermore, citations and reference lists of relevant articles were scrutinized to identify additional pertinent literature.

Ambroxol has demonstrated promising effects in preventing and managing respiratory distress syndrome (RDS). It can enter the placental circulation and rapidly build up in human lung tissue to a much greater extent than in plasma. It promotes fetal lung maturation, surfactant production, and alveolar expansion. Numerous studies have demonstrated the efficacy of antenatal and postnatal ambroxol in the prevention and treatment of RDS. Ambroxol has the potential to be administered intravenously or through nebulization, offering the hopeful possibility of reducing the high failure rate typically associated with non-invasive ventilation in extremely preterm infants, instilling a sense of hope and optimism about the potential of ambroxol. It also shows potential in treating bronchopulmonary dysplasia, meconium aspiration syndrome, and neonatal infections. Ambroxol has been observed to assist in the closure of patent ductus arteriosus in preterm infants by inhibiting vasodilator agents such as nitric oxide and exerting vasoconstrictive properties. However, these biological actions may raise concerns regarding the potential induction of pulmonary hypertension and an increased risk of necrotizing enterocolitis.

The present scoping review also examines the clinical evidence and the potential of ambroxol in reducing the incidence of intraventricular hemorrhage in preterm infants. Ambroxol may have potential analgesic properties in managing neonatal pain, and as it can penetrate the blood-brain barrier, it suggests potential neuroprotective properties. These properties may encompass the modulation of microglial activation and the antagonistic impact on glutamate receptors. Ambroxol’s attributes could contribute to a decreased susceptibility to neurological complications and have demonstrated anticonvulsant effects in preclinical studies.

While low-to-moderate-quality evidence indicates potential applications of ambroxol in neonatal care, further research is needed to determine the drug’s optimal dosing, timing, and safety profiles in this patient population. We need to investigate ambroxol’s potential synergistic effects with antenatal steroids. Exploration is required to assess ambroxol’s potential in reducing the high failure rate associated with non-invasive respiratory support for RDS. Lastly, comprehensive studies on the long-term neurodevelopmental outcomes of neonates exposed to ambroxol are essential.

## Introduction and background

*Adhatoda vasica*, commonly known as Malabar nut, is a well-known medicinal plant in India with a rich history of use in Ayurvedic medicine [1,2]. Its traditional applications encompass a wide range of conditions in both children and adults, including colds, fever, whooping cough, malaria, respiratory tract infections, dysentery, jaundice, gonorrhea, asthma, tumors, pain, wounds, and bleeding hemorrhoids [1,2]. Vasicine, the primary bioactive pyrroquinazoline alkaloid in *Adhatoda vasica*, is the foundation for the semi-synthetic derivative bromhexine and its active metabolite, ambroxol [1-3]. The chemical structure of ambroxol differs from that of bromhexine due to removing a methyl group and adding a hydroxyl group [4,5]. These structural variances render ambroxol capable of exerting additional effects compared to bromhexine, including anti-inflammatory, antioxidant, and local anesthetic properties. Furthermore, ambroxol demonstrates antiviral and antibacterial effects under specific conditions [4,5].

Ambroxol, a cost-effective and versatile drug, is primarily characterized by its mucokinetic properties and mucociliary activity [3,6]. Although the drug has been available since 1973 for treating various respiratory tract diseases in Asia, Africa, the European Union, and South America [3,5-8], it is notably not approved for use in the United States or Canada [9]. While extensive research has been conducted on the clinical efficacy of ambroxol for treating respiratory conditions in adults, its potential application in the prevention and treatment of neonatal diseases has received limited attention [10]. Consequently, neonatal textbooks do not address ambroxol [11,12] or provide only cursory coverage of its use in this context [13,14].

## Review

Objectives of the scoping review

The objectives of this current scoping review are manifold. First, it aims to comprehensively explore and consolidate the empirical data regarding the use of ambroxol in managing health conditions in both preterm and term neonates. Second, it seeks to advocate for an unbiased and thorough assessment of ambroxol. Third, it aims to guide healthcare professionals in evaluating the appropriateness of off-label administration. Lastly, it endeavors to promote the inclusion of ambroxol in future antenatal and postnatal research initiatives to improve the care and outcomes of preterm infants.

Methodology

Key scientific databases such as Google Scholar, PubMed, Cochrane Library, and Europe PMC, as well as electronic versions of relevant pharmacology and neonatal textbooks, were systematically searched to identify pertinent literature on ambroxol in perinatal and neonatal medicine. Gray literature was also meticulously explored using Google. The search utilized key terms without linguistic or temporal limitations until June 2024. The review process only considered the English abstracts for literature written in languages other than English. All types of study designs, encompassing human and non-human studies, randomized controlled trials (RCTs), observational studies, case reports, and reviews, were included in the search criteria. Moreover, the citations and reference lists of relevant articles were scrutinized to identify additional pertinent literature.

Antioxidative and anti-inflammatory properties

Ambroxol exhibits significant antioxidative and anti-inflammatory properties [3,15,16]. Oxidative stress arises from an imbalance between reactive oxygen and nitrogen species and the body’s antioxidant mechanisms [17]. It is prevalent in neonates, especially preterm infants, due to reduced antioxidant defenses and exposure to toxic free radicals [17]. Evidence suggests that oxidative stress and non-infectious inflammation play a role in the pathogenesis of several neonatal diseases, including respiratory distress syndrome (RDS), bronchopulmonary dysplasia (BPD), periventricular leukomalacia (PVL), necrotizing enterocolitis (NEC), patent ductus arteriosus (PDA), and retinopathy of prematurity [17]. However, effective and safe antioxidant or anti-inflammatory agents for routine prevention or treatment of these diseases are currently unavailable [17,18].

Ambroxol’s antioxidative properties include scavenging free radicals and protecting against lipid peroxidation [3,16]. In vitro and in vivo experiments have shown its ability to scavenge hydroxyl radicals, hypochlorous acid, superoxide anion, and hydrogen peroxide [6,16]. Additionally, ambroxol has demonstrated protective effects against reactive oxygen species-induced lipid peroxidation in various human pulmonary disorders and animal models [6]. Moreover, in intoxicated male albino rats, ambroxol attenuated oxidative stress and genotoxicity induced by a neonicotinoid insecticide, as evidenced by reductions in catalase, superoxide dismutase, lipid peroxidation, chromosomal aberrations, and total micronucleated polychromatic erythrocytes [19]. The co-administration of ambroxol attenuated all these oxidative stress and genotoxicity parameters [19].

In models of acute inflammation, ambroxol effectively reduced the release of the lipid mediator leukotriene B4 and decreased levels of pro-inflammatory factors such as nitric oxide (NO), tumor necrosis factor-alpha (TNF-α), interferon-gamma, and several interleukins (ILs) such as IL-6 [15,20]. Ambroxol may also inhibit cell apoptosis in the lung, brain, and other tissues [21-23]. Additionally, ambroxol may inhibit cell apoptosis and increase serum uric acid, an endogenous antioxidant [17,24]. Preclinical studies in adult rat models have demonstrated ambroxol’s protective effects against intestinal, hepatic, and renal ischemia-reperfusion injuries [25-27]. Furthermore, a case study suggested prolonged high-dose ambroxol administration may mitigate hepatic fibrosis in Gaucher’s disease [28].

Antibacterial and antifungal properties

Research conducted in vitro and on animals has demonstrated that ambroxol effectively inhibits biofilms formed by bacteria and fungi [29-33]. This suggests that ambroxol possesses both antibacterial and antifungal properties [34]. Ambroxol has exhibited efficacy against a range of microorganisms, including coagulase-negative staphylococci (CONS) [35], *Pseudomonas aeruginosa* [29,36,37], *Enterococcus faecalis* [33], *Serratia marcescens* [30], *Proteus mirabilis* [31], *Clostridioides difficile* [38], and *Candida albicans* [32,34]. Consequently, it is plausible that ambroxol enhances the action of various antimicrobial agents such as vancomycin, ciprofloxacin, azithromycin, fluconazole, and voriconazole [6,34,37,39-41]. In vitro studies have indicated a significant synergistic effect between ambroxol and vancomycin against coagulase-negative CONS biofilms [35]. Furthermore, in vivo experiments employing antibiotic lock therapy with a combination of ambroxol and vancomycin have demonstrated a substantial ability to eradicate CONS biofilms in a rabbit model of catheter-related bloodstream infections [35]. However, it is important to note that there is a lack of robust clinical evidence supporting the use of ambroxol as an antimicrobial synergist.

Fetal/neonatal lungs

Antenatal prevention of respiratory distress syndrome

Hyaline membrane disease, also known as RDS, results from a deficiency of pulmonary surfactant [14]. Standard care to prevent RDS and neonatal death involves administering antenatal steroids (ANSs) to mothers at risk of preterm birth [42-44]. It is essential to note that ANSs are considered off-label due to the lack of approval from the US Food and Drug Administration [45]. The optimal therapeutic window for ANS administration is between one and seven days [44], but approximately 50% of women deliver outside this window [43,46]. Moreover, 30%-50% of women delivered extremely preterm before completing their ANS course [47-49]. Research suggests that a partial ANS course may be less effective than a complete one, contributing to its relatively low overall efficacy [44,48,50,51]. The overall effectiveness of ANS is relatively low, even when properly administered to mothers who are at genuine risk of imminent preterm delivery [44]. ANS-treated preterm neonates, particularly extremely preterm ones, still exhibit high residual morbidity [44]. The number needed to treat to prevent one case of RDS was 19 among preterm neonates born at 35 weeks gestation or less and 55 in those born at more than 34 weeks gestation [44]. The use of ANS at late preterm gestations is associated with an increased risk of neonatal hypoglycemia [44].

Recent arguments suggest that preterm exposure to ANS may elevate the risk of long-term adverse outcomes for neonates delivered either late preterm or at term [44]. Studies have demonstrated that 40% of mothers given ANSs subsequently delivered at term, and the short- and long-term outcomes of their infants were worse compared to infants born at term to mothers with no corticosteroid administration [52,53]. These infants had an increased risk of serious infections during the first 12 months of life [52], admission to neonatal intensive care units, endotracheal intubation (ETT), smaller head circumference, and neurodevelopmental or behavioral disorders [53]. Others have addressed some of the implications and limitations of these two studies [54]. Nevertheless, the results of these two studies have generated an urge to find a different, safer antenatal treatment for RDS.

Animal studies have shown that ambroxol has potential biological mechanisms for preventing and treating RDS. Ambroxol easily crosses the placental circulation [14]. It is recognized for its significant accumulation in human lung tissue, with concentrations approximately 17-25 times higher than in the plasma [55]. It stimulates type II pneumocyte cells to produce and release surfactant in a cell-specific manner, promoting lung maturation and function in preterm animals [3,6,56]. Ambroxol-treated preterm animals have shown enhancement of lung maturation, alveolar expansion, and lung function [3]. Ambroxol has been shown to increase alveolar phosphatidylcholine levels, promote the storage of lamellar bodies in type II pneumocytes, and induce lysosomal exocytosis [3,57]. It encourages the movement of mucus in the airway and impedes lysosomal phospholipase, the enzyme responsible for breaking down pulmonary surfactant [14]. Ambroxol-treated preterm animals had enhancement of lung maturation, alveolar expansion, and lung function [3]. In addition, it increases the concentration of lamellar bodies in the amniotic fluid of pregnant mothers after oral administration of a 30 mg dose every eight hours [58].

A Cochrane systematic review assessed prophylactic ambroxol versus betamethasone or placebo in women at risk of preterm birth to prevent RDS [59]. The RCTs analyzed in this Cochrane review encompassed infants with a minimum gestational age of 27-28 weeks and a birth weight ranging from 1,540 g to 2,305 g. These RCTs are outdated and, therefore, did not include a comparison between ambroxol combined with ANSs and ANSs administered alone or with a placebo/no treatment. To date, no RCT has been conducted to assess the effectiveness of ambroxol in combination with ANSs compared to ANSs alone. Notably, three RCTs included in this Cochrane review were only published as abstracts, limiting the identification of crucial clinical outcomes. The remaining RCTs did not assess significant neonatal outcomes such as administering pulmonary surfactant, BPD, intraventricular hemorrhage (IVH), or NEC. This Cochrane review categorized the analyzed RCTs as outdated and small-scale, with very low-to-moderate quality and low or unclear risk of bias. The Cochrane review reported a statistically non-significant difference in the risk of RDS between ambroxol and betamethasone (risk ratio (RR) = 0.79, 95% confidence interval (CI) = 0.59-1.07, seven RCTs, 728 women/758 neonates) or between ambroxol and placebo/no treatment groups (RR = 0.74, 95% CI = 0.46-1.20, two studies, 204 women/204 newborns). It also indicated a non-significant reduction in the risk of perinatal mortality between ambroxol and betamethasone (RR = 0.51, 95% CI = 0.23-1.12, six RCTs, 648 women/657 neonates) or between ambroxol and placebo/no treatment (RR = 0.61, 95% CI = 0.19-1.98, one RCT, 116 women/116 neonates). Thus, the Cochrane review determined that there is insufficient evidence to either support or refute the use of ambroxol in women at risk of preterm birth for the prevention of RDS, perinatal mortality, and adverse effects.

In a recent network meta-analysis (NMA), Chinese researchers performed the surface under the cumulative ranking curve (SUCRA) to demonstrate that antenatal ambroxol ranks highest in reducing the incidence of RDS and neonatal death compared to ANSs [60]. While this NMA indicated antenatal ambroxol as potentially the most effective treatment for lowering the incidence of RDS and neonatal death, caution is warranted when solely relying on SUCRA rankings to identify the best treatment, particularly as the quality of evidence diminishes [61,62]. Suggestions have been made to integrate uncertainty into treatment decisions by visually examining CI overlaps of different treatments against a constant comparator [61,62]. The ideal treatment, supported by at least moderate-quality evidence, should exhibit the highest SUCRA with non-overlapping CIs compared to other therapies [61,62]. Assessing the cited NMA in this context reveals challenges in determining the most effective treatment among ambroxol, betamethasone, or dexamethasone, given overlapping CIs and varying levels of evidence quality. Another meta-analysis by Chinese researchers previously recommended antenatal ambroxol over ANSs for mothers at risk of inevitable preterm birth to prevent neonatal RDS [63]. An older study indicated a lower risk of mortality in preterm neonates treated with pre and postnatal ambroxol compared with those treated with postnatal ambroxol only [64].

Preeclampsia affects approximately 6.0% of nulliparous pregnant women and significantly contributes to preterm births, increasing the odds of very preterm (<32 weeks) delivery fivefold compared to women without preeclampsia [65]. Based on theoretical considerations, ambroxol has been proposed as a potentially repurposed medication for managing preeclampsia [10,66]. If confirmed, the use of ambroxol could offer diverse benefits for preterm infants, potentially reducing both the incidence of preterm births and associated complications.

Consistent with these reviews and others [67], further research is necessary to evaluate the short- and long-term neonatal and maternal outcomes of the prophylactic combination of ambroxol and ANSs in women at risk of extremely preterm birth (≤28 weeks gestation). The following research areas require immediate attention: can ambroxol act synergistically with ANSs? Given that approximately 50% of at-risk pregnant women fail to complete the full course of ANSs, is ambroxol a viable supplementary treatment in such instances? Can ambroxol serve as a viable alternative to ANSs for pregnant women for whom ANS is not a feasible option? This is crucial as inadequate ANS treatment can significantly impact neonatal outcomes.

Postnatal treatment of respiratory distress syndrome

Respiratory support and early surfactant replacement therapy (SRT) are considered the standard treatment for RDS [68-72]. Non-invasive ventilation (NIV) is strongly recommended over invasive ventilation through ETT for spontaneously breathing extremely preterm infants with RDS [69-71]. However, one of the main challenges of NIV is its high failure rate, leading to the need for ETT within the first 27 hours of life, which ranges from 38% to 67% in preterm infants born at or before 28 weeks of gestation [47,71,73]. This failure may result in serious consequences such as severe IVH and pneumothorax [71,74]. Surfactant deficiency is the leading factor contributing to the failure of NIV [71]. Consequently, early SRT is crucial to address this failure and mitigate its serious consequences [69,71].

The common methods of SRT include invasive administration via ETT or less invasive delivery via a thin catheter [69,71,75,76]. The challenge lies in achieving fully non-invasive SRT while maintaining NIV [71,77]. Surfactant nebulization is the only non-invasive SRT option; however, it remains an aspiration yet to be fulfilled, largely due to the absence of an approved and commercially available nebulizer for delivering surfactant aerosols [77]. Recent reviews have examined the evidence, pitfalls, and obstacles of nebulized SRT [77,78].

In a 2010 Chinese-language meta-analysis, which included six RCTs and 823 preterm infants, the efficacy and safety of intravenous (IV) ambroxol compared to conventional treatment for RDS in preterm infants were assessed [79]. The conventional treatment included mechanical ventilation, electrolyte management, and acid-base therapy. The meta-analysis found that ambroxol significantly reduced the incidence of RDS (odds ratio (OR) = 0.24, 95% CI = 0.15-0.64, p < 0.01) without causing any adverse events. In this meta-analysis, the effect was estimated using OR. It is worth noting that the OR is larger than the RR, which, if misinterpreted, may lead to overestimating the intervention effect [80]. Notably, this meta-analysis did not reference a small Egyptian placebo RCT that reported positive findings [81]. The Egyptian RCT involved 120 preterm neonates born at 28-34 weeks of gestation. None of the neonates in the study received SRT as it was not available at the study center. The incidence and severity of RDS, the need for and duration of invasive respiratory support, and mortality were significantly lower in the IV ambroxol group compared to the placebo group.

The effectiveness of IV ambroxol in reducing the incidence of RDS, along with RDS-associated complications and mortality, has been supported by several studies. A 2012 Chinese-language systematic review and meta-analysis of 17 Chinese-language RCTs involving 1,732 neonates established the efficacy of IV ambroxol in reducing the burden of RDS [82]. Subsequently, various small RCTs [83-86] and observational studies have further substantiated ambroxol as an effective therapy for RDS [87-89]. A recent small, non-blind, Chinese RCT demonstrated that the combination of surfactant and IV ambroxol proved more effective than the combination of surfactant and budesonide instillation in treating RDS in 90 preterm neonates with a mean gestational age of 30 weeks [89]. Additionally, a Chinese non-blind RCT revealed the superior efficacy of IV ambroxol with high-frequency oscillatory ventilation compared to conventional mechanical ventilation in 73 RDS preterm neonates born at 30-33 weeks of gestation [90]. Furthermore, a small Chinese RCT involving 100 preterm neonates born at a mean gestational age of 34 weeks demonstrated a synergistic effect between surfactant and IV ambroxol [84].

In a recent Chinese-language observational study, the clinical effects of ambroxol in combination with nasal continuous positive airway pressure (nCPAP, n = 60) were compared with ambroxol in combination with bi-level positive airway pressure (BiPAP, n = 60) [87]. The results indicated that the combination of ambroxol and BiPAP was more clinically effective than the combination of ambroxol and nCPAP. This was evidenced by improved arterial blood gas measurements, reduced incidence of BPD, and shortened hospital stays. Furthermore, a published case report highlighted the effective treatment of a 27-week-old female preterm infant weighing 750 g with RDS using intravenous ambroxol in combination with nCPAP [91].

The existing body of evidence strongly supports the utilization of ambroxol for treating RDS and reducing the necessity of SRT and the elevated failure rates associated with NIV. It is important to note that the majority of these studies included preterm neonates born at gestational ages exceeding 28 weeks. As will be elaborated upon, ambroxol can be administered through various pharmaceutical formulations, such as nebulization or IV, thereby warranting further research in this promising and intriguing area.

Bronchopulmonary dysplasia

BPD is a notable complication of prematurity [18,68]. It is a complex condition influenced by pulmonary inflammation, oxidant stress, and ventilator-induced lung injury (VILI) [18,92]. Currently, no antioxidants are available to prevent BPD [18]. Corticosteroids, known for their potent anti-inflammatory properties, have been extensively studied over five decades in numerous RCTs to assess their effectiveness and safety in averting BPD [18,92]. These RCTs have explored various modes of postnatal corticosteroid administration, including systemic, inhalation, and intratracheal instillation of budesonide combined with exogenous surfactant as a vehicle [18,92]. Several systematic reviews of these RCTs have been conducted to compile and assess them [92]. A recent Cochrane overview synthesized and evaluated the evidence from nine published systematic reviews up to April 2023, ultimately advising against routine corticosteroid use [92], in agreement with other systematic reviews, narrative reviews, and clinical guidelines [69,92,93]. However, a recent study revealed increasing rates of postnatal corticosteroid use over the years, underscoring the need for alternative safe therapies for BPD prevention [94].

In 2000, a German double-blind ambroxol-placebo RCT involving 102 preterm neonates with a mean gestational age of 30 weeks who had not received surfactant was published [95]. The study concluded that early IV ambroxol can reduce the incidence of BPD but does not impact inflammatory processes beyond the first week of life. This led to the recommendation of a longer period of ambroxol therapy. A 2010 Chinese-language meta-analysis, based on six RCTs and 823 preterm infants, reported that ambroxol significantly reduced the incidence of BPD with no observed adverse events (OR = 0.41, 95% CI = 0.23-0.75, p < 0.01) [79]. Furthermore, a recent, small, Chinese, non-blind RCT demonstrated that ambroxol effectively reduces lipid peroxidation damage and improves the antioxidant capacity of preterm neonates [88]. Another small Chinese RCT showed that IV ambroxol-surfactant more effectively inhibited an inflammatory environment and oxidative stress than instilled budesonide-surfactant in preterm neonates with RDS [84].

Since 1991, the fetal viability limit in Japan has been 22 weeks of gestation with positive outcomes [96]. Consequently, the Canadian Neonatal Network and the Evidence-based Practice for Improving Quality (EPIQ) visited a prominent advanced neonatal intensive care unit (NICU) in Osaka, Japan [96]. The visit aimed to acquire knowledge from Japanese neonatal practices, replicate successful methods, and stimulate new research initiatives [96]. The 2012 EPIQ publication reported an observed practice in the visited Japanese NICU [96]. The practice involved the administration of ambroxol to stable preterm neonates to reduce pulmonary secretions and the incidence of BPD [96]. However, the EPIQ assessed this practice as lacking evidence-based support [96].

VILI has been identified as a potential risk factor for BPD [18,92]. A study conducted on mechanically ventilated adult rats demonstrated that a high dose of intraperitoneal ambroxol (50 mg/kilogram of body weight, kg) provided protection against VILI [97]. The mechanism of action involved the upregulation of gamma-glutamylcysteine synthetase by ambroxol, leading to increased glutathione production and inhibition of the transcription factor Jun (c-Jun) [97].

A previous observation from two decades ago highlighted the lack of knowledge regarding the long-term neonatal outcomes of ambroxol [98]. Regrettably, this knowledge gap remains. Therefore, immediate attention must be given to large-scale, comprehensive research efforts to address it.

Meconium aspiration syndrome

A systematic review with a meta-analysis was conducted to assess the effectiveness of ambroxol in neonates with meconium aspiration syndrome (MAS) [99]. The abstract of this systematic review was exclusively reviewed due to its publication in the Chinese language. The meta-analysis indicated a substantial variance in cure rates between ambroxol and conventional treatment (RR = 1.29, 95% CI = 1.11, 1.48, 11 RCTs), oxygen therapy (RR = -10.71, 95% CI = -15.87, -5.55), and hospital stay (RR = -3.12, 95% CI = -4.81, -1.43). Additionally, an RCT published in Chinese, which involved 120 neonates with MAS, indicated that the combination of nebulized ambroxol and low-dose dexamethasone was safe and associated with a reduction in the time required for clinical improvement [100]. Furthermore, another small RCT demonstrated the beneficial effects of the ambroxol-budesonide combination for MAS [101]. The authors of both RCTs recommended the routine use of ambroxol in MAS [100,101].

A recent publication detailed a non-blinded RCT conducted in China involving 138 full-term neonates afflicted with MAS [102]. The trial used a machine learning algorithm based on pulmonary ultrasound for MAS diagnosis. It aimed to assess the efficacy and safety of surfactant administration, IV ambroxol, and a combination of both in MAS treatment, with each group comprising 46 neonates. The findings indicated equivalent efficacy between surfactant and ambroxol while underscoring the superior effectiveness of the combined treatment. The study concluded that adding ambroxol to surfactant therapy merits consideration in MAS treatment. The authors acknowledged the study’s limitations, including its small sample size, potential influence of operator proficiency, and environmental factors. They emphasized the necessity for a larger RCT to validate the results.

Pneumonia

In previous discussions, it has been established that ambroxol possesses antibacterial properties. Additionally, co-treatment with ambroxol has been linked to significant elevation in lung tissue and airway surface fluid levels of various antibiotics, such as beta-lactams, glycopeptides, macrolides, nitrofurans, and rifamycins [40]. The 2010 Chinese-language meta-analysis referenced above, involving six RCTS and 823 preterm infants, undertook a comparison of the efficacy and safety of IV ambroxol with conventional RDS treatment in preterm infants, including mechanical ventilation, electrolyte therapy, and acid-base management [79]. This meta-analysis revealed that ambroxol significantly reduced the incidence of pulmonary infection (OR = 0.24, 95% CI = 0.14-0.38, p < 0.01) with no reported adverse events [79].

A small, non-blind, Chinese RCT demonstrated the beneficial effects of inhaled ambroxol as an adjunctive treatment for neonatal pneumonia [21]. Similarly, two other small, non-blind, Chinese RCTs indicated the benefits of IV ambroxol as an adjunctive therapy for neonatal pneumonia [103,104]. A recent meta-analysis of two small Chinese RCTs that evaluated the efficacy of ambroxol-budesonide inhalation for neonatal pneumonia concluded that this combination was more effective than non-combined therapy, with an OR of 1.61 [105].

Patent ductus arteriosus

In the above-cited 2010 Chinese-language meta-analysis (six RCTs, 823 preterm infants), ambroxol administration was observed to significantly reduce the incidence of PDA without any reported adverse effects (OR = 0.33, 95% CI = 0.17-0.67, p < 0.01) [79].

Real-world evidence has illustrated the potential of ambroxol in inducing the closure of a hemodynamically significant PDA (hsPDA) in an extremely preterm infant. Dr. Al-Abdi recently provided care for a female preterm infant born at 26 weeks of gestation. On the fifth day post-birth, the infant was diagnosed with an hsPDA measuring 2.0 mm, leading to substantial left-to-right shunting. Upon follow-up with an echocardiogram on day 30 of life, it was observed that the PDA had reduced to 1.8 mm in size, albeit with persistent significant left-to-right shunting and dilation of the left atrium and ventricle. Initially, the decision was made not to pursue medical or surgical closure of the hsPDA, as the infant was solely on nasal CPAP with 4 cmH_2_O and 21% oxygen. At 33 days of life, the infant received oral ambroxol at 10 mg/kg twice daily for five days as a mucoactive agent. Subsequently, clinical indicators of the hsPDA began to ameliorate. A subsequent echocardiogram confirmed complete closure of the hsPDA and resolution of the left heart-side dilation after the ambroxol regimen.

There is a lack of direct biological research exploring the exact mechanism through which ambroxol closes the PDA. However, it is conceivable that ambroxol may lead to the closure of the ductus arteriosus (DA) either directly or indirectly by influencing the ambient conditions that support the ongoing duct patency in preterm infants. An earlier in vitro study demonstrated that ambroxol can induce vasoconstriction in an isolated rabbit pulmonary artery following noradrenaline stimulation [106]. Therefore, it is plausible to suggest that ambroxol may bring about vasoconstriction in the DA.

Prostaglandin E2 (PE2) is acknowledged as the primary physiological factor responsible for inducing PDA [107,108]. Following the first week of life, other vasodilators (NO, TNF-α, IL-6) assume greater significance than PE2 in instigating PDA. Ambroxol has demonstrated efficacy in reducing the production or activity of these vasodilators [15,20]. Various preclinical studies have presented evidence supporting the ability of ambroxol to decrease NO production and activity [109-113]. The combined administration of an NO synthase inhibitor and indomethacin in premature animals and humans results in significantly enhanced ductus constriction compared to indomethacin alone. Consequently, it is postulated that drugs attenuating the production or activity of NO may serve as a beneficial adjunct in PDA closure. The degradation of PE2 to an inactive metabolite occurs in type II pneumocyte cells [108]. Considering that ambroxol stimulates type II pneumocyte cells [3,6], it is reasonable to propose that ambroxol may enhance the uptake of PE2 into these cells.

The hsPDA poses challenges for both short- and long-term neonatal outcomes [114-117]. Defining hsPDA, determining the optimal approach for its closure, selecting the initial medication, and deciding on the method of administration are all ongoing subjects of debate. Ibuprofen, acetaminophen, or indomethacin are currently the preferred first-line monotherapies for the closure of hsPDA [114], although their use in this context is considered off-label [118]. Notably, the failure rate for hsPDA closure following monotherapy treatment is unacceptably high, ranging from 30% to 60% [114]. Surgical ligation or the insertion of a coil device via cardiac catheterization is the last resort for closing hsPDA after medications have failed [115,119,120]. Invasive PDA closure is associated with poor long-term neurological outcomes and serious post-ligation cardiorespiratory syndrome [115,121]. Thus, alternative therapeutic approaches have been recommended before surgical intervention.

One such approach involves dual hsPDA pharmacotherapy with ibuprofen as the first-line or rescue treatment, with dual therapy offered to preterm infants who do not respond to monotherapy [116,117]. Currently, the evidence supporting dual treatment is confined to two small RCTs and several small non-RCTs [116,117,122,123]. The failure rate of dual therapy is reported to be high [116,117]. Discrepancies regarding the effectiveness of first-line dual treatment stem from the findings of two systematic reviews with meta-analyses [116,117]. The principal cause of this inconsistency is that one systematic review amalgamated RCTs and non-RCTs in a single meta-analysis, while the other conducted separate meta-analyses for RCTs and non-RCTs. Both systematic reviews concluded that the certainty of evidence for dual therapy is low. Another approach suggests administering a second course of ibuprofen or indomethacin and a third course of acetaminophen orally or ibuprofen before resorting to invasive PDA closure [49,115]. This approach is solely based on expert opinion and has not been evaluated in RCTs [115].

It is essential to recognize that ibuprofen, indomethacin, and acetaminophen target specific biological factors associated with PDA (PE2 inhibition). Relying solely on these three medications is linked to a high expected failure rate. Ibuprofen is associated with severe adverse effects, including gastrointestinal perforation, which may result in fatality [115,124]. In the BeNeDuctus trial, the BPD rate was higher in the group treated with ibuprofen compared to the placebo group (50.9% vs. 33.3%) [49]. As a result, the authors of this RCT indicated that closing hsPDA with ibuprofen might pose more harm than the hsPDA itself. They recommended further research into alternative, safer, and more effective treatments. The use of acetaminophen has been linked to an increase in mortality rates in the NICU [125]. The potential advantages of indomethacin have not resulted in improved long-term neurological outcomes [126]. Therefore, it is advisable to research the potential effectiveness of ambroxol in managing hsPDA, considering its unique mechanisms of action. Pending further studies in this area, the potential utilization of ambroxol for the off-label treatment of hsPDA is viable.

Peripheral nervous system

Preclinical research indicates that ambroxol demonstrates an ability to antagonize voltage-gated ion channels, notably Na+1.8 (Nav1.8) and Nav1.7, with greater potency than benzocaine, lidocaine, or mexiletine [5]. Nav1.8 channels, prevalent in nociceptive C fibers within dorsal root ganglion neurons (DRG), are a target of ambroxol [5,127]. Additionally, it has been observed that ambroxol inhibited the Nav1.7 channels, the primary subtype in the DRG of human adults, as well as in nociceptive sensory neurons and sympathetic ganglion neurons [128]. Furthermore, the structural similarity of Ca^2+^ channels to the voltage-gated ion Na^+^ channels and its presence in sensory neurons suggests a potential for ambroxol as a local anesthetic [5]. This is evidenced by its usage in lozenges (20 mg) for alleviating sore throat since 2002 and its potential for topical or oral treatment of neuropathic pain associated with various conditions, such as regional neuropathic pain, complex regional pain syndrome, trigeminal neuralgia, fibromyalgia, and Gaucher’s disease [3,127-130].

Painful procedures, including skin breaks, are frequently performed in NICUs without adequate pain management [131]. The detrimental impact of pain on the neurodevelopmental, behavioral, and cognitive outcomes of premature infants has been well-documented [132]. Therefore, it is recommended that measures aimed at alleviating this pain be considered neuroprotective and capable of reducing the IVH [132]. Surprisingly, the potential analgesic effects of ambroxol in neonatal pain management have not been explored. Considering this potential avenue for future research and clinical application is imperative. However, it is essential to consider that Nav channel subtypes differ between rodents and humans, potentially resulting in differing interactions of ambroxol with these channels and exhibiting subtype preference variations [128].

Central nervous system

Experimental data indicates that ambroxol has demonstrated the ability to readily traverse the blood-brain barrier (BBB), achieving peak concentration in the striatum around 60 minutes following administration of both low and high doses [133]. It is noted that the BBB in neonates may become compromised due to factors such as hyperosmolar load, hypercarbia with acidosis, asphyxia, metabolic acidosis, vasculitis (meningitis), and abrupt elevation in arterial and/or venous blood pressure [132].

Brain development

Serotonin (5-hydroxytryptamine, 5-HT) plays a crucial role in the development of the mammalian central nervous system (CNS) [134]. It supports various fundamental processes of fetal brain development, including neuronal proliferation, differentiation, migration, synaptogenesis, and the expression of neuronal gamma-aminobutyric acid receptors [132]. Disruption of the 5-HT system during early gestation and early development, caused by stress or drug exposure, is linked to altered cognitive ability, neurodevelopmental disorders such as autism spectrum disorders (ASDs), and an increased incidence of psychopathologies such as schizophrenia [132,134]. Among the seven receptor families for 5-HT, the 5-HT3 receptors are the only ligand-gated ion channel receptor [134].

Recent findings indicate that the 5-HT3 receptors emerge as a novel target during the development of the CNS [134]. Ambroxol may act as a 5-HT3 receptor antagonist and an inhibitor of the 5-HT serotonin transport site (SERT) [135]. Consequently, the use of ambroxol during pregnancy may potentially affect cognitive ability or result in neurodevelopmental disorders in the offspring. It is important to note, however, that not all drugs that antagonize the 5-HT system will necessarily alter cognitive ability or induce neurodevelopmental disorders. For example, despite serotonin re-uptake inhibitors exerting their effect by binding to the SERT, their impact on fetal brain development is relatively limited [132,136,137].

No research exists to evaluate the occurrence of neurodevelopmental impairment related to prenatal or postnatal ambroxol use. Nevertheless, it has been observed that the global prevalence of long-term neurodevelopmental impairment in extremely preterm births may be comparable or lower in regions where ambroxol is possibly employed, as opposed to areas where it is unavailable, such as the United States or Canada [138,139]. As this remains speculative, it is crucial to undertake thorough investigations into the neurodevelopment of children exposed to ambroxol prenatally or postnatally. Furthermore, meticulously structured, extensive prospective studies are indispensable for examining the enduring impacts of prenatal and postnatal ambroxol administration on fetal and infant brain development. However, we advise avoiding co-administering ambroxol and selective serotonin reuptake inhibitors during the perinatal period until further guidance is provided.

Intraventricular hemorrhage

IVH is one of the prevalent and severe complications of prematurity [68,140,141]. It manifests very early in the lives of preterm infants, with half of the instances occurring within the first six hours of life [142,143]. A variety of preventive measures are currently being enforced. However, these measures lack robust evidence or have potential adverse effects that may outweigh their benefits [126,144,145].

A 2010 Chinese-language meta-analysis (six RCTs, 823 preterm infants) indicated a significant reduction in IVH incidence with ambroxol administration without any reported adverse events (OR = 0.39, 95% CI = 0.24-0.64, p < 0.01) [79]. The specific biological mechanism by which ambroxol may prevent IVH is not well understood. It is conceivable that ambroxol may diminish the occurrence of IVH in preterm infants by enhancing their overall well-being, potentially through reducing pain stress and the prevention of RDS and PDA, as discussed earlier. Another plausible mechanism by which ambroxol could confer this benefit is its neuroprotective effects, as will be expounded upon in the subsequent section.

As discussed in the PDA section, an earlier in vitro study showed that ambroxol could induce vasoconstriction in an isolated rabbit pulmonary artery following noradrenaline stimulation [106]. Therefore, it is plausible that ambroxol may have a vasoconstrictive effect on cerebral blood flow similar to ANSs [42,146]. Suppose the presumed cerebral blood vasoconstriction caused by ambroxol is validated. Given the current lack of certainty in this area, it is imperative to prioritize comprehensive large-scale research endeavors to assess the impact of vasoconstriction on the developing brains of neonates.

Neuroprotection

Microglia, specialized macrophages in the CNS, play a crucial role in immune response [20,132]. When activated by factors such as hypoxia-ischemia or infection-inflammation, they can release substances such as cytokines and reactive oxygen and nitrogen species, posing a potential threat to premyelinating oligodendrocytes and neighboring differentiating oligodendrocytes or neurons [132]. This microglia activation in white matter is a notable feature in human prematurity encephalopathy [132].

A recent study on an intracerebral hemorrhage model of adult male mice demonstrated that ambroxol at doses of 35 mg/kg and 70 mg/kg mitigated microglial activation, reduced pro-inflammatory cytokines, facilitated neuronal survival, and minimized white matter fiber bundle damage [20]. This effect was attributed to the suppression of endoplasmic reticulum (ER) stress [20]. Therefore, ambroxol could be regarded as an anti-microglial agent and a promising neuroprotective treatment for encephalopathy of prematurity and neonatal hypoxic-ischemic encephalopathy, similar to the effects of melatonin and minocycline [132]. In light of erythropoietin’s declining status as a neuroprotective agent in preterm infants [147], it is imperative to explore ambroxol as a viable alternative for clinical use.

Ambroxol has been found to exhibit antagonist effects on the α-amino-3-hydroxy-5-methyl-4-isoxazolepropionic acid (AMPA) receptors [5,135]. In the realm of neuroprotective pharmacology for stroke management, the exploration of AMPA antagonists as potential drugs has been a subject of interest [5,148]. In a rat model induced with stroke, it was observed that ambroxol led to reductions in stroke volumes, cytotoxic edema, white matter degeneration, and necrosis [22]. These reductions were concomitant with improved behavioral outcomes and changes in both structural and functional connectivity [22]. The proposed mechanism of ambroxol involves the facilitation of recovery in energy metabolism, cellular homeostasis, membrane repair mechanisms, and redox balance [22]. Furthermore, in rats treated with ambroxol for a week following the onset of a stroke, significantly less white matter degeneration of the external capsule and decreased necrosis of the stroke area were evident [22].

Similarly, ambroxol enhanced functional recovery by reducing infarct volume in a mouse model induced with stroke [149]. This study suggests that ambroxol may promote the differentiation of neural stem cells (NSCs) into neurons and interfere with their differentiation into astrocytes by increasing the expression of beta-glucocerebrosidase (GCase) to activate the Wnt/beta-catenin signaling pathway in the penumbra following an ischemic stroke [149]. Additionally, ambroxol has been found to stabilize protein folding in the ER, thereby alleviating ER stress and enhancing overall neuroprotection [23]. Due to its multifaceted mechanisms, ambroxol has been proposed as a promising neuroprotective therapy in acute and chronic hypoxic-ischemic brain injury [23]. It can induce a reduced energy demand phenotype and exert anti-inflammatory and antioxidant effects [23]. Ambroxol has emerged as a potential neuroprotective agent in amyotrophic lateral sclerosis, Parkinson’s disease, and Gaucher’s disease, with ongoing RCTs further investigating its therapeutic potential [9,23,150-154]. Ambroxol has garnered considerable attention in these diseases for its capacity to enhance GCase activity and decrease alpha-synuclein levels [9,22,151,153,154].

Seizure

In vivo studies have demonstrated that ambroxol effectively inhibits recombinantly expressed voltage-gated ion rat Nav1.2 channels [5]. Nav1.2 channels are widely distributed in the CNS, particularly in cortical and hippocampal glutamatergic pyramidal cells [155]. These channels play a significant role in the nodes of Ranvier and the axonal initial segment during the early postnatal development of rats [155]. Recent research indicates that Nav1.2 channels play a critical role in various diseases, including benign familial neonatal/infant seizures, epileptic encephalopathies, ASDs, and other neuropsychiatric conditions [155].

Glutamate, the predominant fast excitatory neurotransmitter in all regions of the CNS, plays a vital role in neuronal signaling [148]. However, glutamate-mediated excitotoxicity can lead to damage in conditions such as hypoxia-ischemia or hypoglycemia, where excessive release and impaired reuptake of glutamate result in the overstimulation of glutamate receptors and subsequent neuronal cell death [132,148]. Among the glutamate receptors, AMPA receptors are ligand-gated ion channels contributing to excitatory neurotransmission [148]. It is noteworthy that during the neonatal period, AMPA receptors are overexpressed, leading to heightened levels of excitatory neurotransmission compared to adults [132].

Glutamate receptor antagonists have demonstrated efficacy in inhibiting seizures across various models, such as seizures induced by electroshock and chemical agents such as pentylenetetrazol [5,156]. Studies have shown significant anticonvulsant effects of ambroxol (20 mg/kg intraperitoneally) in the pentylenetetrazol-induced seizure rat model [157]. Furthermore, ambroxol has ameliorated seizures in Gaucher’s disease [9,154]. However, it is noteworthy that ambroxol syrup was found to induce focal epileptic seizures following oral administration of 30 mg in an 18-year-old female with drug-resistant symptomatic epilepsy [158]. This patient was already on two antiepileptic drugs, namely, levetiracetam (3 g/day) and zonisamide (300 mg/day), and was suspected of having Sturge-Weber syndrome. Possible factors contributing to this outcome include the role of other ingredients in ambroxol syrup and the individual susceptibility of the patient to ambroxol [157,158]. Mechanistically, no causal link between ambroxol and seizure induction has been identified [157]. Notably, perampanel, a non-competitive antagonist of the AMPA-type ionotropic glutamate receptor, has been approved as an adjunctive therapy for focal-onset seizures in patients 12 years and older, with or without secondarily generalized seizures [156].

Clinical safety of ambroxol

Ambroxol is a safe medication for children and adults [159,160]. High-dose IV ambroxol of 1,000 mg/day for five to six days used antepartum in pregnant women to stimulate fetal lung maturation has proven efficacious and safe [6]. Toxicology studies have revealed that ambroxol has a low index for acute toxicology [128]. The 50% lethal dose is around 10,000 mg/kg in rats [128]. Ambroxol did not impair fertility or postnatal development [5]. It was not mutagenic, tumorigenic, or carcinogenic in rodents with high doses of up to 1,000 mg/kg [5]. Despite ambroxol effectively crossing the BBB, no harmful effect was reported, even at high doses [20].

Ambroxol was not embryotoxic and teratogenic at oral doses of up to 3,000 mg/kg in rats and 200 mg/kg in rabbits [3]. The first epidemiological study investigating the risk of major birth defects in women who took ambroxol during pregnancy has just been published in Japan [161]. It was a multicenter prospective cohort study using patients’ data for consultation on the safety of drugs during pregnancy over 20 years. It demonstrated that no significantly increased risk of major birth defects was observed after first-trimester exposure to ambroxol compared to the control group (2.1% (7/341) vs. 1.7% (26/1525), adjusted OR = 1.1, 95% CI = 0.18-7.2, p = 0.88). However, the sample size of the ambroxol group was small, with only 341 pregnant mothers. This Japanese study showed that the proportion of miscarriage was lower in the ambroxol group than in the control group (1.6% vs. 4.2%). Still, the artificial abortion was higher in the ambroxol group (3.2% vs. 1.0%). The authors explicitly acknowledged that the nature of the study precludes further exploring the reason for artificial abortion and why it is more in the ambroxol group. As mentioned in the introduction, ambroxol is the active metabolite of bromhexine, and bromhexine is the semi-synthetic derivative of vasicine [1-3]. Vasicine was found to have activity similar to oxytocin; thus, it may stimulate the uterus and cause abortions via prostaglandin release [1,2]. So, perhaps this was the reason for the higher artificial abortion in the ambroxol group. Because of the paucity of large epidemiological data on ambroxol use during pregnancy, the package insert of ambroxol (Mucosolvan®) currently states that “You should not take this medicine while you are pregnant, especially during the first 3 months.”

Several mild adverse of ambroxol have been reported, including headache, abdominal pain, vomiting, dyspepsia, diarrhea, and exanthem [3,59,160]. Anecdotally, ambroxol has been reported to cause severe allergic reactions (anaphylactic) and severe cutaneous adverse reactions [3,160]. A recent case report has suggested ambroxol was probably the culprit of leukocytoclastic vasculitis in a man [162]. It was shown that two weeks of 30-60 mg daily parenteral ambroxol causes xanthine and calcium oxalate stones in rats [163]. This could be because ambroxol may increase serum uric acid and urine pH [24].

Ambroxol’s vasoconstrictive effects [99] and its potential to reduce NO production or activity [109-113] raise concerns about the possibility of inducing or exacerbating pulmonary hypertension and posing a risk factor for NEC. Nevertheless, it is important to note that ambroxol exhibits anti-inflammatory and antioxidant properties, typically associated with mitigating pulmonary hypertension and NEC. Notably, no direct evidence exists of a causal relationship between ambroxol and NEC or pulmonary hypertension in neonates [59]. However, an epidemiological study on RDS conducted across 65 NICUs and involving 17,192 Italian infants revealed that antenatal ambroxol and ANSs were the established standards for inducing lung maturation in 1995 [164]. Notably, the widespread utilization of ANS commenced worldwide in 1994 [44]. The study documented no increased rate of pulmonary hypertension [164]. It is worth mentioning that pulmonary hypertension was an exclusion criterion in a study on RDS [89].

Pharmaceutical formulations of ambroxol

Ambroxol is marketed in various pharmaceutical formulations, including IV and intramuscular solutions, syrups, granules, tablets, capsules, suppositories, creams, lozenges, oral slow-release formulations, and nebulized solutions [3,4,6,21,165]. Currently, only oral formulary is available in Saudi Arabia.

One of the advantages of ambroxol is that it can be effectively delivered to the lungs using a standard nebulizer [21]. This advantage allows convenient and accessible treatment administration. Nebulized ambroxol was more effective than IV formulation for various respiratory diseases in children [166,167]. A Chinese non-blind RCT showed that nebulized ambroxol was as effective as IV in preventing RDS in 125 preterm infants born between 28 and 37 weeks of gestation [168]. A Chinese-language RCT found that nebulized ambroxol was more effective than IV in treating 120 neonates with MAS [100]. Contrarily, a Chinese non-blind RCT showed that micropump IV infusion of ambroxol was more effective than nebulized ambroxol in treating RDS in 56 preterm neonates born at 28-34 weeks of gestation [88]. The discrepancy between the two studies may be because pediatric patients have more effective spontaneous breathing than preterm ones, and hence, more ambroxol reaches their lungs.

Ambroxol dosage

The optimal dose of ambroxol has not been established. Ambroxol’s half-life is about 10 hours; thus, it can be prescribed twice daily [9]. In adult humans, 30 to 1,000 mg of IV ambroxol produced plasma levels of 0.11 to 2.1 mg/L [5]. Table 1 depicts doses that have been used in various clinical studies. The author prescribes an oral dose of 10 mg/kg of body weight, administered every 12 hours for five days, for newborn infants who can tolerate oral intake.

**Table 1:** Ambroxol dosage. RDS: respiratory distress syndrome

Indication	Dosage	Reference number
Mucolytic	Oral in three divided doses:	[59]
Children 2–5 years	15 to 30 mg/day	
Children 5–12 years	30 to 45 mg/day	
Children 12–18 years	60 to 90 mg/day	
Adults	60 to 180 mg/day	
Antenatal maternal administration for prevention of RDS		[59,60]
Intravenous	1,000 mg every 12 hours for 2 days	
	1,000 mg daily for 2–5 days	
Oral	30 mg every 8 hours for 7–10 days	
	60 mg every 12 hours for 7–10 days	
Neonatal administration for prevention and treatment of RDS		
Intravenous	7.5 mg/kg every 6 hours for 5 days	[95]
	10 mg/kg every 12 hours for 5 days	[81,83]
	15 mg/kg every 12 hours for 5 days	[89]
	15 mg/kg immediately after birth, then 30 mg/kg daily for 2 days	[168]
	30 mg/kg every 12 hours for 3 days	[84]
	30 mg/kg daily for 4–6 days	[85]
	30 mg/kg daily for 7 days	[86]
Nebulization	30 mg/kg daily for 2 days	[168]
Meconium aspiration syndrome: *Intravenous*	7.5 mg/kg daily for 7 days	[102]
Neonatal pneumonia: *Nebulization*	7.5 mg every 12 hours for 7 days	[21]
The author’s oral dose for the neonates who have tolerable oral intake	10 mg/kg every 12 hours for 5 days	

Off-label ambroxol use

Off-label drug use (OLDU) is the unapproved use of approved drugs. It pertains to prescribing pharmaceutical drugs that deviate from the approved indications, ages, dosages, and dosage forms specified by the country’s regulatory agency [118,169]. OLDU is prevalent due to the lack of regulatory control over medical decision-making post-market approval [169-172]. This practice is particularly widespread in perinatal-neonatal care as pregnant women and neonates are typically underrepresented in RCTs [169-172]. Thus, the predominant utilization of pharmaceutical drugs in perinatal-neonatal care often involves off-label usage [169-172]. This encompasses medications such as ANSs, indomethacin, ibuprofen, and acetaminophen [45,118]. Additionally, the use of ambroxol in perinatal-neonatal care is regarded as off-label.

OLDU is legal and does not imply that pharmaceutical drug is contraindicated or disapproved [169,172,173]. Physicians are not legally obligated to inform their patients about the status of a drug’s label [118,173-175]. However, there is ongoing debate regarding whether specialized informed consent, such as enhanced therapeutic consent [176], should be obtained due to the limited knowledge about the prescribed medication [118,173-175]. OLDU has its advantages and disadvantages [172,177]. It allows for innovation in clinical practice, especially when approved treatments have failed or are contraindicated [177]. However, it presents an ethical dilemma for physicians seeking autonomy in prescribing drugs that match individual patient needs, regardless of their label status, or depriving patients of potential therapeutic benefits [169,173,177]. It also raises concerns about the thorough evaluation of drug safety and efficacy, undermines the incentives for manufacturers to conduct rigorous studies, and may discourage evidence-based practice [172,177].

OLDU indicates insufficient data available to grant approval status and the risks or benefits of using pharmaceutical drugs in a particular situation have not been thoroughly examined [169,173]. The use of pharmaceutical drugs for purposes not approved by regulatory authorities is considered acceptable when it prioritizes the patient’s best interests and is supported by scientific evidence demonstrating the safety and efficacy of the prescribed drug [118,172-175,177]. Without such evidence, OLDU should be by expert consensus or practice guidelines [173-175,177]. This approach ensures that OLDU is conducted consistently with the highest standards of care [173-175,177]. Several checklists have been developed to ensure the safe implementation of OLDU [172]. As part of its development process [178]. Ambroxol has successfully completed preclinical studies and phases I through III of clinical trials. The supporting evidence for prescribing ambroxol in perinatal-neonatal care is low to moderate, as per the categorization by Largent et al. [176], the off-label use of ambroxol is considered suppositional OLDU.

Discussion

The primary aim of the present comprehensive scoping review is to promote an impartial and equitable examination of ambroxol, specifically within the realm of prenatal and postnatal care. We need to consider the cumulative weight of the clinical evidence while acknowledging any limitations or areas that require further investigation. We also recognize that anecdotal reports can provide valuable insights, even if they are subjective.

The resurgence of interest in the mucolytic agent ambroxol has shed light on its diverse therapeutic applications, particularly in neonatal medicine. As research continues to unveil the multifaceted pharmacological properties of ambroxol, its potential as a versatile therapeutic option for a range of neonatal conditions has become increasingly evident.

Ambroxol has exhibited promising efficacy in the prevention and management of RDS. It can permeate the placental circulation and accumulate rapidly in human lung tissue to a notably greater extent than in plasma [55]. Ambroxol facilitates fetal lung maturation, surfactant production, and alveolar expansion in preterm animals [3,6,56]. Additionally, it augments the concentration and storage capacity of lamellar bodies, thereby promoting lysosomal exocytosis while inhibiting the enzyme responsible for the breakdown of pulmonary surfactant [3,14,57,58].

Multiple RCTs have demonstrated the effectiveness of antenatal ambroxol in preventing RDS. However, evidence from a Cochrane review remains inconclusive [59]. A network meta-analysis has suggested that ambroxol may be a more effective option than ANSs for RDS prevention [60]. Several small-scale clinical studies conducted in China, the Middle East, and South America have also indicated the efficacy of ambroxol in treating RDS [81,83-89]. A systematic review further supports these findings, underscoring the potential of ambroxol as a viable RDS treatment [79]. Administering ambroxol both before and after birth may prove more effective in reducing neonatal mortality compared to antenatal administration alone [64]. Ambroxol can be administered intravenously or through nebulization [21,83], offering the potential to reduce the high failure rate commonly associated with NIV in extremely preterm infants. Due to its anti-inflammatory, antioxidant, and anti-biofilm properties against microorganisms, ambroxol has demonstrated efficacy in treating BPD [79,95]. It is an adjunct therapy for MAS and neonatal infections, particularly pneumonia [79,99-104].

The research indicates that ambroxol holds promise in facilitating PDA closure in preterm infants [79]. This effect is attributed to ambroxol’s ability to inhibit vasodilator agents such as NO, TNF-α, and IL-6 [15,20], thereby promoting PDA closure. Additionally, ambroxol’s vasoconstrictive properties may contribute to the direct closure of PDA [106]. These findings shed light on the potential use of ambroxol in managing PDA in preterm infants.

Ambroxol has demonstrated potential for reducing the incidence of IVH in preterm infants [79]. The biological mechanism through which ambroxol exerts its protective effect against IVH remains incompletely elucidated. It is plausible that ambroxol’s ameliorative impact on IVH may be attributed to its capacity to improve overall infant well-being, potentially by mitigating the pain stress and the incidence of RDS and PDA. Additionally, ambroxol’s neuroprotective properties or its potential to induce vasoconstriction are conceivable mechanisms underlying its beneficial impact in preventing IVH.

It is important to note that the majority of research on ambroxol in perinatal-neonatal medicine has focused on preterm neonates born at gestational ages exceeding 28 weeks. This population represents a significant target for reducing complications associated with extreme preterm birth. Most RCTs on antenatal ambroxol are old and performed in the European Union States. Similarly, evidence on ANS is based on old RCTs from a high-income population [44]. Meanwhile, it is acknowledged that much of the current evidence supporting the use of ambroxol in neonates originates from studies conducted in China, as shown above. This may raise questions about the level of trust in research from China and lead to potential biases or dismissal of the available data. Medical research misconduct is indeed common in Chinese academia [179]. Medical research articles from China or co-authored by Chinese investigators constituted most retractions in the last three years [180,181]. Thus, the integrity of Chinese research has come under growing scrutiny and criticism [180,181]. Moreover, an old animal American study did not support the notion that antenatal ambroxol can improve preterm lung functions [182].

With careful clinical implementation and robust scientific evidence, ambroxol may emerge as a valuable tool in the comprehensive management of neonatal health and well-being. However, it is essential to acknowledge that the drug’s optimal dosing, timing, and safety profiles in this patient population require further investigation. Accordingly, prescribing ambroxol for perinatal-neonatal conditions may be considered suppositional OLDU in countries where ambroxol is approved for use as an expectorant. Moreover, the vasoconstrictive effects of ambroxol, as well as its potential to decrease NO production or activity, raise concerns regarding the potential to induce or exacerbate pulmonary hypertension and pose a risk factor for NEC.

The local anesthetic effect of ambroxol is significantly greater than lidocaine’s due to its antagonistic action on multiple sodium and calcium voltage-gated ion channels in nociceptive fibers [5,128]. However, its potential analgesic properties in managing neonatal pain have never been investigated. Ambroxol has generated significant interest in the neonatal context due to its neuroprotective properties. It has demonstrated the ability to modulate microglial activation and display antagonistic effects on AMPA glutamate receptors [5,135]. These combined actions, alongside ambroxol’s antioxidant and anti-inflammatory properties [3,16], may contribute to reducing the risk of neurological complications, including cerebral hemorrhage, stroke, prematurity-related encephalopathy, and hypoxic-ischemic encephalopathy [22,23,149]. Moreover, ambroxol has been observed to exert anticonvulsant effects in preclinical studies [157].

One should consider that ambroxol may function as a 5-HT3 receptor antagonist and as an inhibitor of the serotonin transport site [135]. Consequently, it can potentially harm brain development and function, counteracting the positive effects associated with ambroxol. Speculatively, it can be inferred that ambroxol is deemed safe, as evidence suggests that regions where ambroxol is utilized exhibit overall lower rates of long-term neurodevelopmental impairment in extremely preterm infants compared to areas where ambroxol is inaccessible. We recommend against the concurrent administration of ambroxol and selective serotonin reuptake inhibitors during the perinatal period until further guidance is issued.

A superb, carefully designed, large, and intensive RCT was recently published in the New England Journal of Medicine. Unfortunately, its results were disappointedly negative. It found that high-dose IV erythropoietin from birth for a period of six to nine weeks administered to extremely preterm infants born at 240-276/7 weeks of gestation did not result in a lower risk of severe neurodevelopmental impairment or death at two years of age [147]. Despite these negative results and expensive, invasive treatment of erythropoietin, professor Joseph J. Volpe, a pioneer in the neurology of newborns, commented on this RCT that “the book erythropoietin should not be closed but should raise the possibility that the duration of erythropoietin treatment was too brief” [183]. Ambroxol is an excellent alternative neuroprotective agent that should be looked at.

It is important to approach the evaluation of ambroxol with an open and unbiased mindset. The key elements of this fair consideration should include a thorough examination of the clinical evidence, a careful review of the study designs, methodologies, and participant characteristics, and data analysis of the clinical trials on ambroxol, regardless of their geographic origin. We must consider patients’ and healthcare providers’ testimonials regarding their experiences with ambroxol. The scientifically rigorous approach to address concerns about the available evidence is not to dismiss this existing evidence but to conduct an additional well-designed RCT that would address the identified concerns and help clarify the situation [184]. Regrettably, the European Patent Office Register Application number EP3145491 for ambroxol has been assigned to a small company with limited financial resources to conduct large RCTs [9]. This has raised concerns about the potential need for industry-sponsored RCTs, given the low cost of ambroxol and the subsequent minimal return on investment. We and other stakeholders have recognized this issue [185].

Several promising future research directions are evident regarding the use of ambroxol in perinatal-neonatal care. These include investigating its potential synergistic effects when used in combination with ANSs, as well as its viability as an alternative option for pregnant mothers who are not suitable candidates for ANSs. A potential area for research involves investigating the potential of ambroxol in mitigating the high failure rate associated with non-invasive respiratory support for RDS. Furthermore, it is essential to explore the potential benefits of ambroxol in various neonatal lung pathologies, its role in treating hsPDA, and neonatal infections.

The scoping review has identified several potential research opportunities for further investigation. The local anesthetic effect of ambroxol is significantly greater than lidocaine’s due to its antagonistic action on multiple sodium and calcium voltage-gated ion channels in nociceptive fibers. Despite this, the potential analgesic properties of ambroxol in the management of neonatal pain have not been thoroughly investigated. Acknowledging and exploring this uncharted territory for future research and clinical application efforts is essential. Investigating ambroxol’s efficacy as a neuroprotective agent and its long-term effects on neonatal outcomes warrants comprehensive evaluation through large-scale, appropriate future research initiatives. Furthermore, comprehensive studies on the neurodevelopment of children who have been prenatally or neonatal exposed to ambroxol are imperative. It is essential to recognize that most studies on ambroxol from a perinatal-neonatal perspective have not adequately encompassed extremely preterm infants, warranting further research.

## Conclusions

As supported by preclinical research, ambroxol exhibits biological plausibility for treating various neonatal conditions. Low-to-moderate clinical evidence suggests its efficacy in preventing or treating RDS, PDA, IVH, and BPD. Additionally, it may have potential as an adjunctive therapy for MAS and neonatal infections, particularly pneumonia. Preclinical investigations indicate that ambroxol may act as a neuroprotective agent, while clinical evidence indicates its local analgesic effects. Notwithstanding its potential benefits, theoretical concerns exist regarding its potential to exacerbate pulmonary hypertension, pose a risk factor for NEC, and antagonize 5-HT receptors involved in brain development. Given the need for further research on ambroxol in perinatal-neonatal medicine, its prescription may be considered suppositional OLDU in countries approved for use as an expectorant.

The use of ambroxol in perinatal-neonatal care presents several potential areas for future research. It is noteworthy that existing studies focusing on ambroxol from a perinatal-neonatal perspective have insufficiently included extremely preterm infants, necessitating further investigation. Research opportunities involve exploring potential synergistic effects when ambroxol is administered concurrently with ANSs, as well as its suitability as an alternative option for pregnant mothers who are unsuitable for ANSs. Furthermore, investigating ambroxol’s potential to reduce the high failure rate associated with NIV respiratory support for RDS is crucial. Additionally, evaluating the potential benefits of ambroxol in various neonatal lung pathologies and its effectiveness in treating hsPDA and neonatal infections are important research avenues. Examining the local anesthetic effect of ambroxol on neonates is also essential. Moreover, comprehensive studies on the neurodevelopment of children who have been exposed to ambroxol prenatally or neonatally, along with an evaluation of ambroxol’s efficacy as a neuroprotective agent and its long-term impact on neonatal outcomes through large-scale, well-designed future research initiatives, are imperative.
